# A Retrospective Analysis of Postmortem *Salmonella* Dublin Cases in Dairy Cattle in British Columbia

**DOI:** 10.1155/2024/9461144

**Published:** 2024-05-27

**Authors:** Ellen Boyd, John Dick, Christine Millar, Kazal Ghosh, Gitanjali Arya, Chelsea Himsworth

**Affiliations:** ^1^ Ministry of Agriculture and Food Government of British Columbia Abbotsford BCCanada; ^2^ School of Population and Public Health The University of British Columbia Vancouver BCCanada; ^3^ Greenbelt Veterinary Services Chilliwack BCCanada; ^4^ World Organisation of Animal Health Reference Laboratory for Salmonellosis National Microbiology Laboratory at Guelph Public Health Agency of Canada Guelph ONCanada

## Abstract

*Salmonella* Dublin is a bovine-adapted bacterial pathogen that primarily affects dairy cattle. The incidence of *S*. Dublin has been increasing across North America, including strains that are multidrug resistant. In British Columbia, the Ministry of Agriculture's Animal Health Center (AHC) reported an increase in cases since 2015, warranting an investigation into how *S*. Dublin is spreading within the province. The objectives of this study were to make use of historical data collected from dairy farms across the province to (1) describe *S*. Dublin cases diagnosed at the AHC between 2007 and 2021, (2) identify risk factors for *S*. Dublin transmission across British Columbia dairy farms, and (3) identify any potential biases associated with passive laboratory-based data that may apply to our results. We found that *S*. Dublin cases diagnosed at the AHC have been increasing over time. Over half of the cases had respiratory symptoms; however, clinical signs tended to be highly variable. The prevalence of respiratory symptoms was mirrored by florfenicol treatment and was suggested to be due to using a first-line antibiotic for more common causes of pneumonia when presented with an *S*. Dublin case. Calves were 38 times more likely to have *S*. Dublin when compared to adults (odds ratio = 38.43, confidence interval = 7.26–203.64), and given the sample population (postmortem cases), it is reasonable to conclude clinical disease is most severe in this age group. Farm premise accounted for a large amount of variability within our model (92% of unexplained variance), suggesting that farm-level management practices may be the most important risk factor for *S*. Dublin infection. In total, only 54% of BC dairy farms submitted to the laboratory between 2007 and 2021; however, proximity to the laboratory did not appear to influence submissions as proportionally; farms within the Fraser Valley submitted as frequently as farms from other regions. We strongly suggest that future work explore factors associated with farm management practices, given our findings regarding the clustering by premises.

## 1. Introduction

*Salmonella* Dublin is a bovine-adapted bacterial pathogen that primarily affects dairy cattle [1]. Once *S*. Dublin enters a herd, it can spread quickly, often resulting in calf loss and abortions, but it can have an asymptomatic stage during initial infection [1]. Once present on farms, it can be difficult to eliminate as many animals become lifetime chronic carriers, resulting in intermittent bacterial shedding [1]. *S*. Dublin bacteria are shed via feces, milk, urine, saliva, and vaginal secretions, with feces containing the highest concentration of bacteria [2]. Individuals can become infected through direct oral contact with contaminated milk, food, water, and/or environmental exposure through manure and/or contaminated equipment [2]. One Danish study estimated that on free-stall farms, the gross margin losses from *S*. Dublin could be as high as $480 CAD/stall [3]. Additionally, *S*. Dublin has the potential to infect humans through contact with infected animals or contaminated dairy and meat products, resulting in illness (bacteriemia), prolonged hospitalization, and, in some instances, death [4].

Currently, the available research regarding risk factors for *S*. Dublin has been conducted in Europe (Denmark, Sweden, and the Netherlands) and Africa (Algeria). The studies have focused primarily on farm-level risk factors (i.e., open herd practices, herd size, proximity to other farms), with some also investigating risk factors at the individual cow level (i.e., breed, age, feed type). The role of biosecurity practices that limit the introduction of new animals (open vs. closed herd) into the herd remains unclear; some work indicates that having an open herd increased the odds of transmission (odds ratio (OR) = 2.0–5.6), but other work reports reduced odds in open herds (OR = 0.06) [5, 6, 7, 8]. Increasing herd size appears to be a risk factor, with ORs increasing by 1.03–1.2 per 100 animals [6, 7, 8, 9]. Regional discrepancies among countries regarding breed as a risk factor have also been reported [5, 7]. Brown Swiss cows appeared more vulnerable to *S*. Dublin infection in Algeria (OR = 15.6, confidence interval (CI) = 1.7–146.2), but Jersey cows were shown to have slightly higher odds in terms of mortality from *S*. Dublin in Denmark (OR = 3.3, CI = 2.7–4.1) [5, 7]. Additional risk factors include region/location, poor biosecurity, organic farming, the density of neighboring herds, and season [5, 6, 7, 9, 10, 11, 12, 13, 14, 15]. Given the variable results among countries, it is important to investigate individual and farm-level risk factors in Canadian dairy herds.

The incidence of *S*. Dublin has been increasing across North America, including strains that are multidrug-resistant [4]. The British Columbia Ministry of Agriculture's Animal Health Center (AHC), the primary diagnostic laboratory for animal health within the province, reported an increase in cases per year since 2015, warranting investigation into how *S*. Dublin is spreading within the province. Thus, the aims of this study were to make use of historical data collected from dairy farms across the province to (1) describe *S*. Dublin cases diagnosed at the AHC between 2007 and 2021, (2) identify risk factors for *S*. Dublin transmission across British Columbia dairy farms, and (3) identify any potential biases associated with passive laboratory-based data that may apply to our results.

## 2. Materials and Methods

### 2.1. Data Collection

All postmortem dairy cases from 2007 to 2021 were pulled from the medical record software (VADDS) at the AHC for individual evaluation (*n* = 1463). Cases (*n* = 12) were excluded from evaluation if tissues were taken from an individual that was not euthanized/deceased (i.e., biopsy of tissue during c-section) or if the case was outside of British Columbia. All remaining cases were evaluated by a registered veterinary technician and then audited by a veterinarian and/or veterinary pathologist. Data extracted from the case records included submission date (month, year), farm location, veterinarian, farm name, veterinary clinic, signalment (breed, sex, age), clinical signs, treatments given, diagnoses, and bacterial culture results. A small percentage of the cases included additional data, i.e., feed type, herd size, and number of sick individuals, but these data were not included in the final analysis due to the high proportion of missing values. *S*. Dublin cases were determined to be either primary or secondary causes of morbidity and mortality based on the pathologist's interpretation, which is summarized in the diagnosis field of the case record. Primary cases of *S*. Dublin were ones where the lesions associated with *S*. Dublin infection were sufficient to account for the morbidity and mortality of the individual. A secondary case was one in which *S*. Dublin-associated lesions were present; however, another pathogen or disease process was the most likely primary driver of morbidity and mortality. It should be noted that, in British Columbia, *S*. Dublin is a notifiable disease, meaning that all suspect cases must be reported to the Ministry of Agriculture within 24 hr [16]. There were no reports other than those made by the AHC.

### 2.2. Sample Processing

The AHC is an accredited laboratory within the American Association of Veterinary Laboratory Diagnosticians, with postmortem examinations adhering to procedural standardizations. All postmortems were performed by a veterinary pathologist, with tissues collected for bacteriology and histopathology. All bacteriology is overseen by a board-certified veterinary microbiologist. Processing tissues from postmortem first involves searing the surface of tissues with a hot spatula and then cutting the surface with a sterilized scalpel. As part of routine bacterial isolation, a sterile swab is used to collect the sample, and then it is inoculated into Columbia Blood agar and MacConkey agar plates (Oxoid, Ontario, Canada) and incubated aerobically for 24–48 hr. To isolate *Salmonella*, the sample is enriched in selenite broth at 42°C for 24 hr and then inoculated onto Hektoen and XLT4 agar (Oxoid, Ontario, Canada) and incubated at 35°C for 24–48 hr. Any typical Salmonella colonies are confirmed by MALDI-ToF MS (Bruker, Ontario, Canada), and *Salmonella* serogroups are determined by slide agglutination testing. If *Salmonella* serogroup D is identified, the sample is sent to the Public Health Agency of Canada for serotyping by whole genome sequencing using SISTR (The *Salmonella* In Silico Typing Resource) and reported according to White–Kauffmann–Le Minor (WKL) scheme [17, 18].

### 2.3. Statistical Analysis

The outcome variable was *S*. Dublin status (positive or negative). Cases were classified as positive if *S*. Dublin was identified through either routine aerobic or selective *Salmonella* culture. Descriptive statistics were used to describe the prevalence, clinical signs, diagnoses, and antimicrobial use within the *S*. Dublin-positive group of cases. Newly infected premises (i.e., positive farm with no previous positive submission within our timeframe) were displayed graphically.

For risk factor analyses, explanatory variables that were considered included season (September–November = fall; December–February = winter; March–May = spring; June–August = summer), region (Fraser Valley; Okanagan; Vancouver Island; Creston; Bulkley Valley), age (Fetus; 0–14 days = neonate; 0.5–5 months = calf; 6–12 months = young heifer; >1 year = older heifer/adult), and breed (Holstein; Jersey; Ayrshire; Brown Swiss). Cases that did not include data for all covariates were excluded. A total of 1,331 cases remained for analysis.

To identify risk factors associated with *S*. Dublin status, the distribution of the explanatory variables was examined among the entire sample of farms included in the final dataset, as well as separately by status (i.e., positive and negative farms). A generalized linear mixed model (GLMMs) was used to examine relationships between *S*. Dublin infection and each of the explanatory variables while controlling for potential clustering by submitting veterinarian and farm. The intraclass correlation coefficient (ICC) was calculated to determine how much variance within the model was explained by the random effect. The initial analysis was performed on all cases where *S*. Dublin was detected, including those deemed only a secondary or incidental cause of mortality. A sensitivity analysis was performed by excluding cases where *S*. Dublin was not the primary cause of mortality. All statistical analyses were conducted using R (R Development Core Team, Vienna, Austria).

### 2.4. Spatial Analysis

To look for clusters in relation to farm proximity (based on latitude and longitude coordinates for each farm) a spatial analysis was conducted. Latitude and longitude were determined using R (R Development Core Team, Vienna, Austria), where farm address was converted into coordinates. This information was imported into SaTScan (Boston, USA) for cluster analysis using a space-time Bernoulli model and scanned for areas with unusually high or low rates of *S*. Dublin by year using a circular window with a maximum spatial cluster size of 50% of the population at risk. The unit of analysis was farm submission per calendar year. For example, if a farm submitted two positive cases within the 2007 year, this would be denoted as one case; however, if a farm submitted one positive case every year for 3 years (2007, 2008, and 2009), this would be denoted as 3 cases. In the later example the cases would only be represented once on the map. Clusters having a corresponding *p* value < 0.05 were visualized in ArcGIS.

### 2.5. Examining Biases from Laboratory-Based Data

Submissions to the AHC were contrasted against registered dairy farms (*n* = 472) within the province as of 2021 regarding the region and examined with a chi-square analysis to determine differences (where *p*  < 0.05).

## 3. Results and Discussion

### 3.1. Results

Ninety of 1,331 (6.8%) dairy postmortem cases submitted to the AHC between 2007 and 2021 were positive for *S*. Dublin. The majority of infected farms (80/90, 88.9%) were located in the Fraser Valley region of the province, and cases were relatively evenly distributed throughout the seasons (Table 1). The vast majority of submissions came from cattle identified as Holstein (1,251/1,331, 94%) (Table 1). Fetuses (445/1,331, 33%) were the most common age group, followed by calves, neonates, and older heifers/adults, which all made up about 20% of submissions, respectively (Table 1). Chi-square or Fisher's exact tests show no significant difference in *S*. Dublin status regarding farm region or breed. There was a significant difference in *S*. Dublin status in terms of age category (*p*-value = < 0.001).

**Figure 1:**
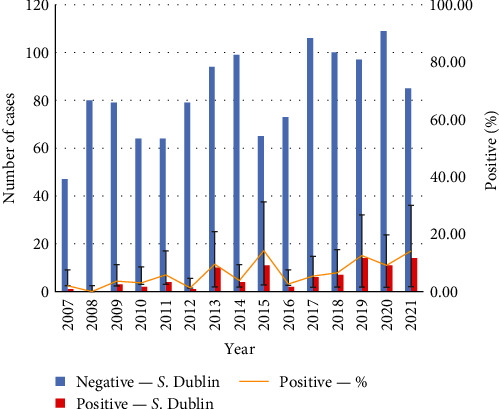
Number and proportion of *S*. Dublin cases submitted to the Animal Health Centre, Ministry of Agriculture, British Columbia, between 2007 and 2021.

**Figure 2:**
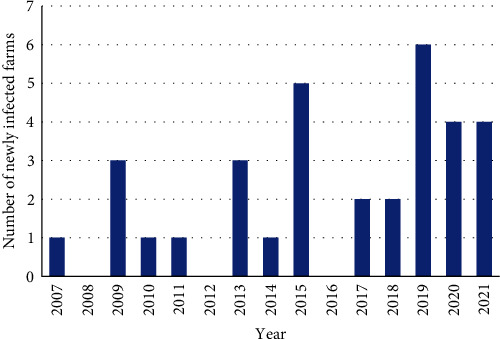
Number of newly detected *S*. Dublin-infected farms (i.e., farms that did not have *S*. Dublin cases in previous years within the timeframe of the study) based on cases submitted to the Animal Health Centre, Ministry of Agriculture, British Columbia, between 2007 and 2021.

Of the 1,331 cases analyzed, there were 90 (7%) positive for *S*. Dublin, with the absolute number of cases (1–90) and percentage of total cases (2%–14%) increasing through time from 2007 to 2021 (Figure 1). In total, 33 individual farms tested positive from 2007 to 2021, with the number of newly infected farms appearing to increase over time (Figure 2). All premises with *S*. Dublin positive cases submitted a positive case in 2 or more years, excluding the new cases from 2021.

*S*. Dublin was the primary cause of morbidity and/or mortality in 84.4% (76/90) of cases. In 26.3% (20/76) of cases where *S*. Dublin was the primary cause of morbidity or mortality, a secondary pathogen was identified. These pathogens included *E. coli* (7/76, 9.2%), *Pasteurella multocida* (3/76, 3.9%), *Mannheimia haemolytica* (3/76, 3.9%), *Streptococcus* sp. (3/76, 3.9%), and *Trueperella pyogenes* (3/76. 3.9%). In cases where *S*. Dublin infection was considered secondary or incidental (14/90, 15.6%), the primary pathogens found most frequently were *Mycoplasma bovis* (6/14, 42.9%), *Histophilus somni* (4/14, 28.6%) and *Mannhemia haemolytica* (3/14, 21.4%). Antimicrobial therapy was reported in 60% of *S*. Dublin cases (Table 2). Florfenicol (46.3%) was the predominant antimicrobial, followed by tulathromycin (20.4%) and TMS (13%). Enrofloxacin was used in 7.4% of cases.

Among cases where *S*. Dublin was the primary cause of morbidity and mortality, the most common clinical signs were respiratory distress (35/76, 46.1%), herd mortality (35/76, 46.1%), and gastrointestinal symptoms (32/76, 42.1%). Symptoms tended to vary by age category, with respiratory symptoms seen predominantly in the calf (22/32, 68.6%) and young heifer (3/4, 75%) group and gastrointestinal symptoms more frequently in the neonate group (13/18, 72%). Less frequent symptoms included sudden death (i.e., death within 24 hr of symptom onset or death after no symptoms) (16/76, 21.1%), ADR (i.e., lethargy, inappetence, wasting, etc.) (14/76,18.4%), abortion (14/76, 18.4%), pyrexia (9/76, 11.8%), neurologic symptoms (3/76, 3.9%), joint swelling (1/76, 1.3%), and petechia (1/76, 1.3%). On postmortem examination, morphological diagnoses for cases where *S*. Dublin was the primary cause of mortality included septicemia (often with interstitial pneumonia) (60/76, 78.9%) and enterocolitis (20/76, 26.4%).

The GLMM that controlled for clustering in relation to the veterinary clinic did not indicate that the veterinary clinic had any impact on the risk of *S*. Dublin infection (random effect = 0). The GLMM that controlled for clustering in relation to farm estimated variance for the random effect of farm was 38.45 (SD = 6.2). The calculated ICC showed that 92% of the unexplained variance within the model was due to farm, indicating that farm of origin impacts on *S*. Dublin status. In the GLMM, the odds of positive *S*. Dublin status were increased in the calf (OR = 38.43, CI = 7.26–203.64) and young heifer (OR = 10.01, CI = 1.5–68.1) groups (Table 3). All other variables did not have statistically significant findings and/or lacked power. A sensitivity analysis was performed comparing models where *S*. Dublin was the primary pathogen only with models where secondary pathogen cases were included, and they were not significantly different.

**Figure 3:**
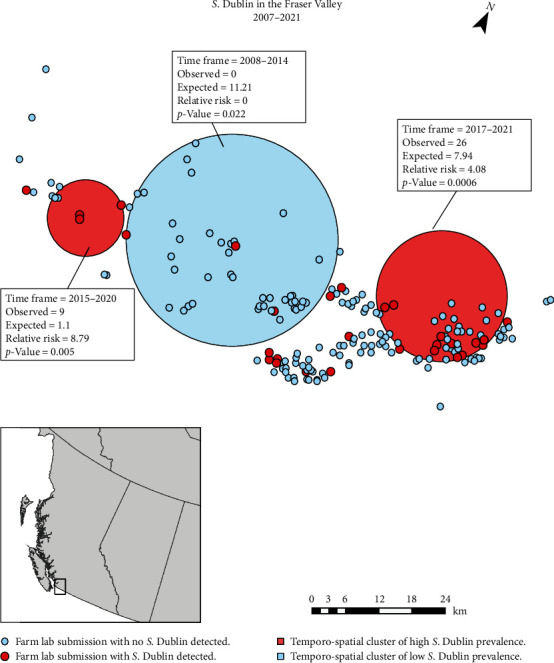
Distribution of dairy farms in British Columbia that have submitted cattle to the Animal Health Centre, BC Ministry of Agriculture for Postmortem examination between 2007 and 2021. The large circles indicate geographic areas with a greater or lesser number of *S*. Dublin cases than expected within the indicated time frame. The unit of analysis was farm submission per year. For example, if a farm submitted two positive cases within 1 year, this would be denoted as one case; however, if a farm submitted one positive case every year for 3 years, this would be denoted as three cases. The areas of high and low risk only apply to the labeled timeframe. In the latter example, the cases would only be represented once on the map. Underlay of the map has been removed to preserve producer privacy.

The geographic clustering of cases was evident only within the Fraser Valley, containing areas of high and low cases (Figure 3). Clusters of higher-than-average cases occurred through 2015–2021, with clusters of lower-than-average amounts of cases occurring earlier from 2008 to 2014. In the high cluster areas, the relative risk of *S*. Dublin infection was 8.79 (*p*-value = 0.005) and 4.08 (*p*-value = 0.0006), respectively (Figure 3).

As of 2021, there were 467 dairy farms within British Columbia, among which 251 (54%) had submitted at least one case to the AHC between 2007 and 2021. There were no differences between the locations of farms that did or did not submit cases to the AHC (Table 4).

### 3.2. Discussion

Overall, based on the historical data collected between 2007 and 2021, the number of *S*. Dublin cases detected at the AHC has increased from 2% to 14.4% of all postmortem dairy cases (Figure 1). *S*. Dublin newly infected premises appear to be increasing as well (Figure 2). As *S*. Dublin is known to persist on farm for a prolonged period cumulative trends may be the more accurate metric to follow to better estimate disease prevalence within the province as some carriers will continue to shed for many years [19]. However, given that only 54% of dairy farms in BC submit cases to the AHC, we speculate that the reported number of *S*. Dublin cases is a gross underestimation of the absolute cases of *S*. Dublin throughout the province.

With the exception of fetuses and neonates, the most common clinical signs among cases where *S*. Dublin was the primary cause of morbidity and mortality were respiratory in nature as a result of sepsis-induced interstitial pneumonia, which is consistent with other studies [20]. One study did find that diarrhea was the most common clinical sign reported, however, did note that their sample population was limited to postmortems that requested *Salmonella* culture [21]. This discrepancy may be due to the nonspecific nature of *S*. Dublin's clinical presentation, and therefore, many respiratory cases may not be requesting *Salmonella* culture specifically. Interestingly, the most common antibiotic used in the *S*. Dublin-positive cases was florfenicol. Although this antibiotic treatment is not effective for the treatment of *S*. Dublin, it is a common treatment for other cases of calf pneumonia (i.e., *Mannheimia*, *Pasteurella*, *Histophilus*, and *Mycoplasma*), suggesting that *S*. Dublin cases may be easily mistaken for other respiratory diseases, such as enzootic pneumonia [22, 23]. Unfortunately, *S*. Dublin is resistant to many “first line” antibiotics, with recent data from the Michigan State University Veterinary Diagnostics Laboratory showing complete resistance to ampicillin, florfenicol, and tetracycline [22]. Only TMS and enrofloxacin have shown any promise in treating *S*. Dublin, but these were only used in 7.4% of the *S*. Dublin cases included in this study. This is likely due to reluctance on the part of veterinarians in using antibiotics like enrofloxacin empirically and highlights the need for an accurate and rapid antemortem test for *S*. Dublin—a test that is not currently available in Canada. Of note, in Canada, extra-label drug use of enrofloxacin is not prohibited in food and animal medicine as it is in the United States [24].

*S*. Dublin was the primary cause of morbidity and mortality in 84.4% of cases where the bacterium was detected. Secondary disease processes were identified in 26.3% of these cases, and these processes were highly variable in nature. Cumulatively, this suggests that *S*. Dublin is an important primary pathogen and does not occur as part of a disease complex. It has been suggested that *S*. Dublin is able to establish and colonize individuals with normal, healthy microbiota due to a virulence plasmid, which allows it to survive when phagocytized [20].

The only significant risk factor for *S*. Dublin infection was age, with calves (0.5–5 months) being at the highest risk. While others have failed to identify this age group as a risk factor, differences in methodology may explain some of these discrepancies. For instance, an Algerian study did not include cases collected from cattle less than a year [5]. A study of asymptomatic *S*. Dublin carriers found that heifers were 11 times (OR = 11, CI = 1.9–63.8) more likely to become chronic carriers of *S*. Dublin when infected, as opposed to calves (OR = 1.2, CI = 0.4–3.4) [15]. This suggests that the risk factors for *S*. Dublin infection and carriage may differ from those associated with *S*. Dublin-related morbidity and mortality.

Our study failed to provide evidence that breed is a risk factor. We, however, acknowledge that we failed to have sufficient power to test this factor and thus strongly encourage future research to investigate this, particularly given that previous work has reported that breed, specifically Brown Swiss, was identified as a risk of *S*. Dublin infection (OR = 15.7, CI = 1.7–146.2) [5]. In line with the majority of previous work, season was not identified as a risk factor in the current study [7, 10, 12]. However, there is some work that suggests that periods of heat stress may result in a higher number of outbreaks, thus increasing the likelihood of positive milk serology in the fall, given the lag time to produce antibodies after infection [11]. Our work did not support this but requires future study to verify our findings given that we were only able to report a tendency, with spring and summer having decreased risk when compared to the Fall reference category (OR = 0.75 and OR = 0.9).

There was no clustering of cases by the veterinary clinic; however, farm of origin accounted for 92% of the variability within the model. This is consistent with research in Denmark that found farm management practices, specifically calving pen management, were important risk factors for *S*. Dublin [20]. A tool created to evaluate these management practices was used in the Danish eradication program, resulting in a drop in infected herds from 26% to 6% in 6 years [25].

Farm region was not associated with the odds of *S*. Dublin positivity; however, within regions, there were clear temporospatial clusters of higher and lower-than-expected risk. Farm density has previously been identified as being positively correlated with the risk of *S*. Dublin infection [7, 9, 10, 11, 13]. However, in our study, there were areas of high farm density with lower-than-expected numbers of cases and vice versa. This suggests that spatial trends require further study as there are likely other key factors that have yet to be identified that contribute to increased relative risk outside of proximity. Additional covariates worthy of further study include similar visiting service providers, mutual heifer rearing, pasture sharing, and/or proximity to standing water.

Interestingly, despite previous studies that have found that proximity to a diagnostic laboratory significantly influences the likelihood that cases will be submitted to that laboratory, we found no significant association among dairy producing region and laboratory submission. That being said, only approximately half of dairy farms submit cases to the AHC, which is the only laboratory offering dairy postmortem services in the province. This may suggest that other factors outside of distance influence why a farm would choose to submit cases or not. One of these factors may be the specific herd health veterinarian or clinic, as 75.7% of all dairy cases were submitted by only three clinics.

One important limitation of this study is its reliance on passive surveillance data; therefore, we cannot be certain of the degree to which our findings are representative of all *S*. Dublin cases across the province. Given that it is legally mandated that anyone suspecting *S*. Dublin infection report to the Ministry of Agriculture, the Ministry's laboratory records may be more representative compared to nonreportable or -notifiable diseases [16]. An additional limitation was the paucity of farm-level data in the laboratory records (e.g., herd size, management practices, etc.), which limited our ability to identify herd- or farm-level risk factors. Finally, it is not possible to validate the information provided on the laboratory submission form; thus, it is possible things like antimicrobial use and symptoms may not be accurate. However, given the goal of submitting an individual for postmortem examination is to determine the cause of death, there is the motivation of the submitter to provide complete and accurate information on the submission form.

## 4. Conclusions

This study suggests that the number of *S*. Dublin infections in BC may be increasing over time and that the pathogen is predominantly causing significant clinical disease in calves less than a year of age. The available case reports suggest that symptoms for *S*. Dublin are primarily respiratory in origin and that a lack of antemortem diagnostic tools can result in misdiagnosis and/or ineffective empirical therapy. There are geographic and temporal clusters of higher risk, but further studies are needed to understand this finding, as well as farm-level risk factors, given the strong clustering of positive cases by farm.

## Figures and Tables

**Table 1:** Characteristics of *S*. Dublin status among postmortem dairy cases (*n* = 1,331) submitted to the Animal Health Centre, Ministry of Agriculture, British Columbia.

Category	Subcategory	Total (%)	Positive (%)	Negative (%)
		*n* = 1,331	*n* = 90	*n* = 1,241
Season	Fall	385 (29.0)	28 (31.1)	357 (29.0)
	Winter	357 (26.8)	26 (28.9)	331 (27.0)
	Spring	288 (21.6)	16 (17.8)	272 (22.0)
	Summer	301 (22.6)	20 (22.2)	281 (22.6)

Farm region	Fraser Valley	1,197 (89.9)	80 (88.9)	1,117 (90.0)
	Okanagan	84 (6.3)	6 (6.7)	78 (6.3)
	Vancouver Island	31 (2.3)	4 (4.4)	27 (2.2)
	Creston	3 (0.2)	0 (0)	3 (0.2)
	Bulkley Valley	16 (1.2)	0 (0)	16 (1.3)

Age	Fetus	445 (33.4)	17 (18.9)	428 (34.5)
	Neonates	269 (20.2)	19 (21.1)	250 (20.1)
	Calf	274 (20.6)	47 (52.2)	227 (18.3)
	Young Heifer	83 (6.2)	5 (5.5)	78 (6.3)
	Older Heifer/adult	260 (19.5)	2 (2.2)	258 (21.0)

Breed	Holstein	1,252 (94.1)	87 (97.0)	1,165 (94.0)
	Jersey	68 (5.1)	2 (2.2)	66 (5.3)
	Brown Swiss	7 (0.5)	1 (1.1)	6 (0.4)
	Ayrshire	4 (0.3)	0 (0)	4 (0.3)

**Table 2:** Distribution of antimicrobials used in *S*. Dublin cases.

Antimicrobial	Total (%)
	*n* = 54
Florfenicol	25 (46.3)
Tulathromycin	11 (20.4)
TMS	7 (13.0)
Enrofloxacin	4 (7.4)
Gamithromycin	3 (5.6)
Tilmicosin	2 (3.7)
Ceftiofur	2 (3.7)
Oxytetracycline	1 (1.9)
Sulfamethazine	1 (1.9)

**Table 3:** Adjusted odds ratios for positive *S*. Dublin status.

Category	Subcategory	Adjusted for clustering on clinic and farm	
		OR^1^	95% CI
Season	Fall	Ref^2^	—
	Winter	0.80	0.38–1.65
	Spring	0.64	0.30–1.38
	Summer	0.79	0.38–1.65

Farm region	Fraser Valley	Ref^2^	—
	Okanagan	0.47	0.01–16.35
	Vancouver Island	1.09	0.00–20,245.69
	Creston	0	0-NA^3^
	Bulkley Valley	0	0-NA^3^

Age	Older Heifer/adult	Ref^2^	—
	Fetus	3.10	0.55–17.15
	Neonates	8.28	1.55–44.32
	Calf	38.43	7.26–203.64
	Young Heifer	10.01	1.50–68.09

Breed	Holstein	Ref^2^	—
	Jersey	1.07	0.13–8.69
	Brown Swiss	7.52	0.00–117,717.12
	Ayrshire	0.00	0.13–8.69

^1^Odds ratio (OR) with 95% confidence interval (CI). ^2^Reference category. ^3^Insufficent power to make an estimate.

**Table 4:** Comparing proportions of BC farms by region and lab use by region.

Region	Number of farms	Proportion	Lab use by region	Proportion	*p*-Value^1^
	*n* = 467		*n* = 251		
Fraser Valley	322	0.69	195	0.78	0.99
Okanagan	82	0.18	28	0.11	—
Vancouver Island	38	0.08	18	0.07	—
Creston	8	0.02	3	0.01	—
Bulkley Valley	17	0.04	7	0.03	—

^1^Chi-square test used to generate *p*-value.

## Data Availability

The data used in this study contains confidential and identifying information about dairy producers in British Columbia, and thus, access to data is restricted given the ethical concerns and veterinary-client-patient privacy.
